# Clinicopathological Factors Influencing Survival After Trimodality Treatment in Non-Metastatic Esophageal Cancer: A Retrospective Single-Center Study

**DOI:** 10.3390/jcm15124635

**Published:** 2026-06-15

**Authors:** Murat Yakin, Nilufer Bulut, Tanju Kapagan, Sevcan Genc, Ulviye Oflas, Sercan Yuksel, Gokmen Umut Erdem

**Affiliations:** 1 Department of Medical Oncology, Basaksehir Cam and Sakura City Hospital, 34480 Istanbul, Turkey; muratyakin@outlook.com (M.Y.); drsevcn@gmail.com (S.G.);; 2 Department of Medical Oncology, University of Health Sciences, 34668 Istanbul, Turkey; 3 Department of General Surgery, Basaksehir Cam and Sakura City Hospital, 34480 Istanbul, Turkey; drsercanyuksel@gmail.com

**Keywords:** esophageal cancer, chemoradiotherapy, survival

## Abstract

**Background**: In locally advanced squamous cell esophageal cancer, concurrent chemoradiotherapy (CRT) is the standard of care, as it especially improves local control and overall survival compared to radiotherapy alone. In contrast, treatment strategies for esophageal adenocarcinoma often parallel those used in gastric cancer, particularly regarding systemic therapy. **Objectives**: This study aimed to evaluate the clinicopathological factors affecting event-free survival (EFS) and overall survival (OS) following trimodality treatment in patients with non-metastatic esophageal cancer. **Methods**: A total of 155 patients diagnosed with esophageal cancer between March 2019 and November 2025 were retrospectively analyzed. Response to concurrent chemoradiotherapy was assessed via thoracic magnetic resonance imaging and endoscopic biopsy. **Results**: Clinicopathological analysis showed that male sex, the presence of lymphovascular invasion, adenocarcinoma histology, poor pathological response and advanced-stage tumors were significantly associated with worse EFS (all *p* < 0.001). In multivariate analysis, stage IVa disease was identified as an independent predictor of both mortality and relapse, with an approximately five-fold increased risk of death (*p* = 0.028) and relapse (*p* = 0.019). Patients with squamous cell carcinoma had a longer median EFS compared to those with adenocarcinoma (18 vs. 8.4 months, respectively). The 3- and 5-year OS rates were 59.2% and 56% in patients with squamous cell carcinoma, compared with 40% and 26% in those with adenocarcinoma, respectively. **Conclusions**: Survival outcomes were more favorable in patients with squamous cell histology and those diagnosed at an early stage. Active surveillance may be considered in selected patients with a complete clinical response to avoid the perioperative mortality associated with surgery.

## 1. Introduction

Treatment strategies for esophageal cancer are primarily determined based on tumor subtype and anatomical location. Chemoradiotherapy (CRT) represents the preferred treatment modality for esophageal squamous cell carcinoma (SCC) due to the higher radiosensitivity of SCC and favorable responses observed with combined modality treatment. In contrast, distal esophageal adenocarcinomas are more commonly managed with perioperative chemotherapy, particularly the FLOT (fluorouracil, leucovorin, oxaliplatin and docetaxel) regimen, which has demonstrated improved survival outcomes [1].

The CROSS trial highlighted the benefit of neoadjuvant chemoradiotherapy in both histological subtypes [2,3]. Neoadjuvant concurrent CRT followed by surgical resection has been shown to improve both disease-free survival (DFS) and overall survival (OS) in patients with locally advanced esophageal cancer [2,4,5]. The CheckMate-577 trial led to the incorporation of adjuvant nivolumab into current treatment guidelines for patients with residual disease following surgery [6]. Patients receiving neoadjuvant CRT in this trial achieved higher pathological complete response rates, with particularly pronounced benefits observed in the SCC subgroup [7].

The current multidisciplinary management of locally advanced esophageal cancer therefore incorporates tumor histology, location, patient performance status, and surgical eligibility when selecting treatment strategies. In selected patients who achieve a clinical complete response after CRT, nonoperative management approaches such as active surveillance or a watch-and-wait strategy may also be considered following multidisciplinary evaluation, particularly in frail or elderly patients [8,9].

The aim of this study was to evaluate the impact of histopathological and clinical characteristics associated with relapse or recurrence on event-free survival (EFS) and overall survival (OS) in patients with early-stage or locally advanced esophageal cancer treated with concurrent CRT and chemotherapy.

## 2. Materials and Methods

This study included 155 patients diagnosed with SCC or adenocarcinoma of the esophagus who received treatment between March 2019 and November 2025 for early-stage or locally advanced disease. Demographic and histopathological characteristics, neoadjuvant and adjuvant treatments, treatment responses, surgical procedures, and pathological staging were retrospectively recorded.

Diagnosis was established through endoscopic biopsy, and clinical staging was performed using thoracic computed tomography (CT) or magnetic resonance imaging (MRI), along with positron emission tomography CT (PET-CT) scans. For tumors in the middle to lower esophagus, concurrent CRT (CCRT) consisted of a total radiation dose of 41.4 Gy delivered in 23 fractions, administered concurrently with weekly carboplatin (area under the curve: 2) and paclitaxel (50 mg/m^2^) over a 5-week treatment period. CRT was followed by restaging endoscopy performed 4–6 weeks after the completion of treatment. Patients with advanced clinical stage or residual wall thickening and those deemed fit for surgery underwent resection. The Ivor Lewis procedure was employed for distally located tumors, while the McKeown procedure was used for upper and middle esophageal tumors, with both approaches including lymphadenectomy. Pathological staging was performed according to the TNM classification system [10]. Resection margins were classified as R0 when the microscopic margin was ≥1 mm and R1 when <1 mm.

Histopathological tumor regression was graded (TRG) based on the proportion of viable tumor cells in the resected specimen, with scores defined as TRG 1 (no viable tumor), 2 (1–10% residual tumor), 3 (11–50% residual tumor), or 4 (>50% residual tumor) [11]. pT0N0 results were classified as complete responses.

Evaluation at 4–6 weeks post-CRT included thoracic MRI and endoscopy. Patients who exhibited no wall thickening on MRI, no mucosal irregularity or dysplasia/malignancy on biopsy, and only mild scarring on endoscopy were re-evaluated at 12–18 weeks with repeat endoscopy. In the absence of disease progression or suspicious lymphadenopathy, a complete response was confirmed, and patients were followed up with at 3-month intervals. Patients who declined endoscopic follow-up or had suspicious biopsy findings despite a clinical response or suspected lymph node involvement without distant metastasis were referred for surgery.

EFS was defined as the time from initiation of treatment to the first instance of relapse or recurrence. OS was defined as the time from diagnosis to death.

### 2.1. Inclusion Criteria

The included patients met the following criteria: the presence of resectable middle–distal esophageal SCC or adenocarcinoma staged as clinical T1–T4 and N0–N3, without distant metastasis and the receipt of platinum-based chemotherapy and/or radiotherapy or perioperative FLOT chemotherapy in the neoadjuvant or adjuvant setting.

### 2.2. Exclusion Criteria

Exclusion criteria consisted of the presence of metastatic disease, incomplete or missing clinical data, and histological subtypes other than squamous cell carcinoma or adenocarcinoma.

### 2.3. Statistical Methods

Statistical analyses were conducted using SPSS for Windows v22.0 (SPSS Inc., Chicago, IL, USA). The Shapiro–Wilk test was used to assess the normality of variable distributions. The Cox proportional hazards model was applied for both univariate and multivariate analyses, with the results reported as hazard ratios (HRs) and 95% confidence intervals (CIs). Variables found to be statistically significant in univariate Cox analysis were included in the multivariate model. The Mann–Whitney U-test was used to compare the non-normally distributed variables of the two groups. Moreover, survival curves were estimated using the Kaplan–Meier method. The factors identified in the univariate analysis (*p* < 0.15) were entered into the Cox regression analysis with backward selection to determine independent predictors of survival. The p-T and p-N variables were not included in the multivariate test.

## 3. Results

The mean age of the 155 patients included in this study was 60.1 ± 12 years (Table 1).

Among the 155 patients included in the study, 129 (83.2%) had squamous cell esophageal carcinoma. According to the clinical stage distribution, 14 patients (9.0%) had c-Stage 1 disease, 65 (41.9%) had c-Stage 2, 46 (29.7%) had c-Stage 3, and 30 (19.3%) had c-Stage 4a disease. In line with the study design, patients with cN0–3 and c-Stage 1–4a squamous cell carcinoma were intentionally included, as the treatment strategy was consistent across these stages. Accordingly, all 129 patients (83.2%) with squamous cell carcinoma received concurrent chemoradiotherapy with paclitaxel and carboplatin, without stage-based differences in management.

The remaining 26 patients (16.8%) had adenocarcinoma. Of these, 13 patients (8.3%) received neoadjuvant FLOT therapy, whereas the other 13 patients (8.3%) underwent upfront surgery without neoadjuvant treatment.

Adjuvant therapy was administered to 17 patients (14.8%): 2 received nivolumab, 1 received a docetaxel–cisplatin–fluorouracil regimen, 1 received a docetaxel–fluorouracil-based FLOT regimen, and 13 received 5-fluorouracil or capecitabine, oxaliplatin, and leucovorin (FOLFOX or XELOX).

In our cohort, 32 patients achieved a clinical complete response confirmed by endoscopy. Of these, 16 (10.3%) did not undergo surgery and were managed with active surveillance, while 16 (10.3%) underwent surgical resection.

The watch-and-wait strategy was applied to patients with initially locally advanced esophageal squamous cell carcinoma who demonstrated no residual disease on endoscopic evaluation, negative biopsy findings, and no suspicious lymph node involvement following the completion of chemoradiotherapy. Eligibility for the watch-and-wait protocol was determined by the multidisciplinary oncology board and was preferentially considered for patients who refused surgery and those who were frail and/or older than 65 years. In the non-surgical group, repeat endoscopic biopsy was performed in patients with suspicious findings, whereas biopsy was omitted in patients with normal mucosal appearance and no evidence of stricture. These patients subsequently continued routine surveillance consisting of scheduled endoscopic examinations and thoracoabdominal CT imaging. During follow-up, two patients developed recurrence and one died (Table 2).

**Figure 1:**
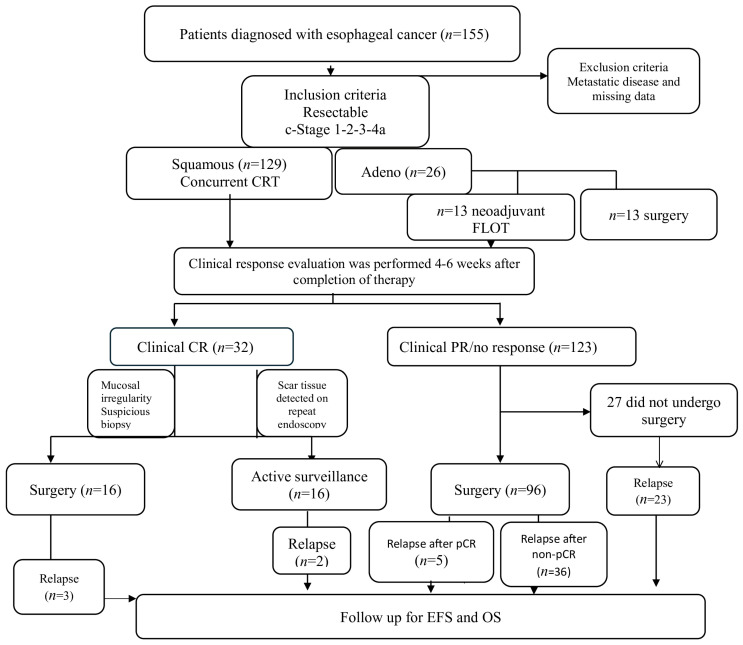
Study profile.

In the non-surgical group, a second endoscopic biopsy was performed in patients with suspicious findings. For those with normal mucosa and no evidence of stricture, biopsy was omitted. These patients continued with routine surveillance, which included scheduled endoscopic evaluations and thoracoabdominal CT imaging. Of the remaining 16 patients who underwent surgery despite clinical complete response, three experienced relapse and two died. Over a median follow-up of 41 months, there was no significant difference in EFS between surgical and non-surgical patients, and the median OS was not reached (*p* = 0.952 and *p* = 0.955, respectively). Among the 43 patients (27.7%) with partial or no response who did not undergo surgery, 23 (18.6%) had a poor clinical response and experienced disease progression during follow-up despite treatment (Figure 1).

Univariate analysis showed that the following factors were significantly associated with poor EFS: male sex (*p* < 0.001), ECOG PS 2-3 (*p* = 0.037), adeno histology (*p* < 0.001), surgical intervention (*p* = 0.001), R1-R2 resection (*p* < 0.001), the presence of lymphovascular invasion (LVI) (*p* < 0.001), tumor grade 3 (*p* < 0.001), pathological T stage 3-4 (pT3-4) (*p* < 0.001), pathological node status (pN1-3) (*p* < 0.001), and pathological stage II–IVa (*p* < 0.001) (Table 2). Multivariate analysis identified male sex as an independent predictor of poor EFS (HR; 2.42, 95% CI: 1.22–4.83; *p* = 0.011), while pathological tumor stage (pT3–pT4a) demonstrated a trend toward poorer EFS without reaching statistical significance (HR; 1.95, 95% CI: 0.90–5.22; *p* = 0.052, Table 3).

**Figure 2:**
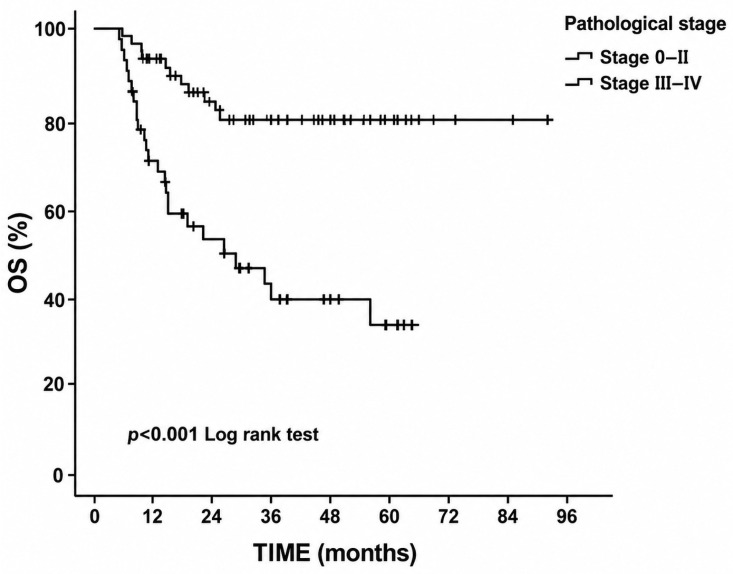
Kaplan–Meier curve of overall survival according to pathological stage.

Univariate analysis also identified several factors significantly associated with poor OS, including male sex (*p* = 0.012), BMI ≥ 19 (*p* = 0.015), ECOG performance status 2–3 (*p* = 0.006), adeno histology (*p* = 0.004), surgical intervention (*p* < 0.001), pathological response score 2–3–4 (*p* = 0.012), tumor grade 3 (*p* < 0.001), the presence of LVI (*p* < 0.001), resection margin R1–R2 (*p* = 0.001), tumor stage pT3–T4 (*p* =0.001), pN1–3 status (*p* < 0.001), and pathological stage III–IVa (*p* < 0.001, Figure 2). Multivariate analysis showed that pathological tumor stage (pT3–pT4) demonstrated a trend toward poorer overall survival, although the association did not reach statistical significance (HR; 2.20, 95% CI: 0.80–6.20; *p* = 0.055, Table 4).

**Figure 3:**
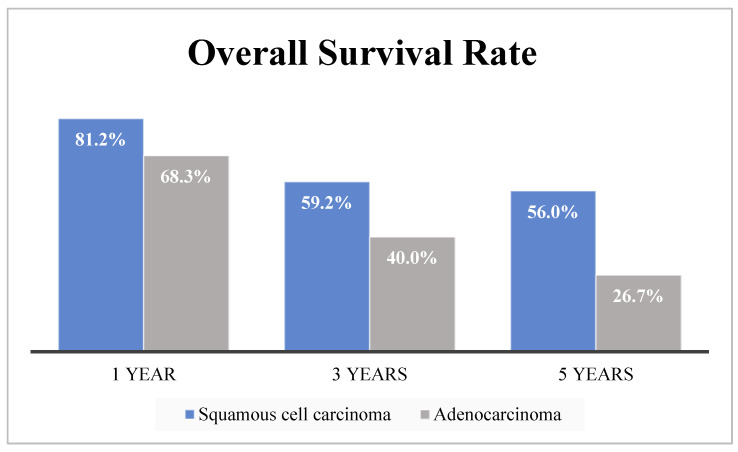
Percentage distribution of overall survival according to subgroups.

In our study, the 1-, 3-, and 5-year overall survival rates were 81.2%, 59.2%, and 56.0% for squamous cell carcinoma and 68.3%, 40.0%, and 26.7% for adenocarcinoma, respectively (Figure 3).

**Figure 4:**
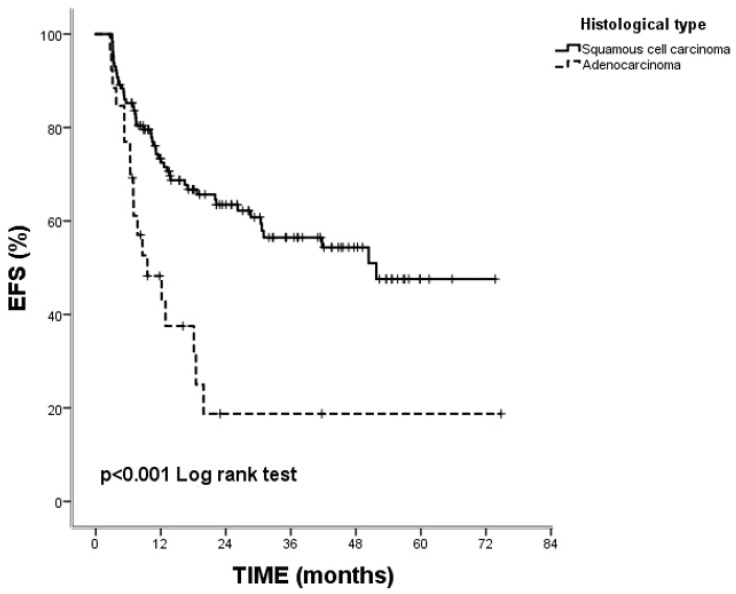
Comparison of event-free survival curves in squamous cell carcinoma and adenocarcinoma by the Kaplan–Meier method.

**Figure 5:**
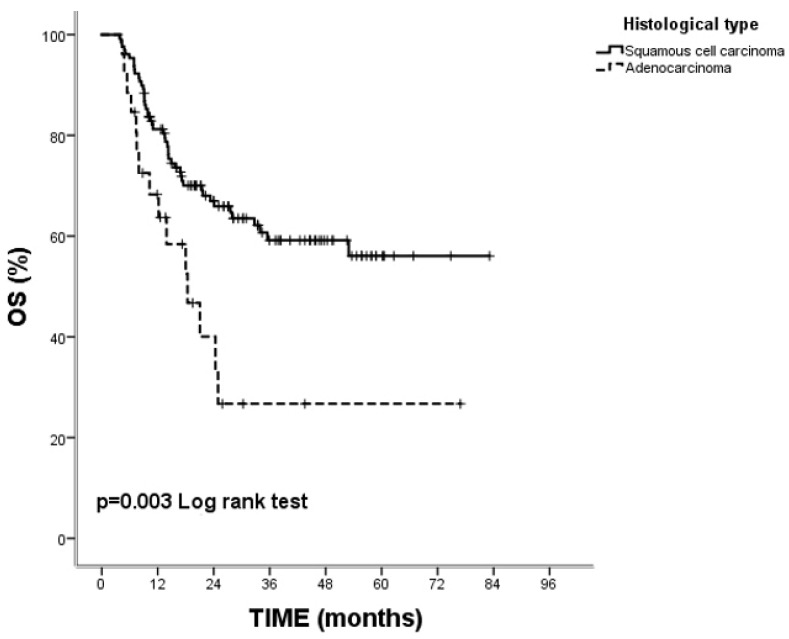
Comparison of overall survival curves in squamous cell carcinoma and adenocarcinoma by the Kaplan–Meier method.

Disease recurrence occurred in 69 patients (44.5%), while 86 patients (55.5%) remained event-free. At the time of analysis, 93 patients (60%) were living and 62 (40%) had died. The median EFS and OS were 15 (range: 5.5–74 months) and 21 months (range: 6–83 months) in the entire cohort, respectively. The median event-free survival was 18 months for squamous cell carcinoma, while it was 8.4 months for adeno carcinomas (*p* = 0.004). While overall survival rates were not obtained in the squamous cell subgroup, a rate of 12 months was observed in the adenocarcinoma subgroup (Figure 4 and Figure 5).

## 4. Discussion

In the CROSS trial, patients who received CRT followed by surgery derived greater benefit from trimodal therapy than those treated with surgery alone, particularly among those with N1 disease. The median OS was 48 months in the trimodality group vs. 24 months in the surgery-only group.

The absence of residual tumor in both the primary lesion and lymph nodes represents the most favorable pathological outcome. In the present study, the pathological complete response rate (TRG 1) was 35.7%, while TRG 2, 3 and 4 were observed in 27%, 17%, and 19.6% of patients, respectively [5]. Among patients undergoing surgery after the standard 4–6-week interval, TRGs 1–4 were reported in 26%, 34%, 22%, and 17% of cases, respectively. When stratified by histologic subtype, TRG 1 was achieved in 55% of patients with SCC compared to 21% of those with adenocarcinoma [3]. No significant differences in relapse-free survival (RFS) or overall survival were observed between patients with a pathological complete response (ypT+N0 or ypT0N+) and those achieving overall complete response (ypT0N0) [12]. In the CROSS trial, adenocarcinoma histology and relatively earlier-stage disease (mainly cT3N1) predominated, and treatment consisted of platinum-based concurrent chemoradiotherapy. The reported pCR rate was 29%.

In our cohort, 129 of 155 patients had squamous cell carcinoma; among them, 41 (45.6%) achieved pT0, 59 (65.6%) were pN0, and 29 (32.2%) achieved a complete pathological response (ypT0N0). In comparison, among 26 patients with adenocarcinoma, 2 (8.7%) achieved pT0, 8 (34.8%) were pN0, and 2 (8.7%) achieved ypT0N0. The higher prevalence of squamous cell carcinoma in our series may be attributed to the predominance of N0–N1 disease (94%) and early-stage tumors (65.5%), which are known to respond more favorably to trimodality therapy.

Regarding overall survival, pathological stage III–IVa was associated with a 2.2-fold increased risk of death, showing borderline statistical significance (*p* = 0.055). In contrast, stage IVa disease was associated with a 5.2-fold increased risk of death, which was statistically significant (*p* = 0.028). In the literature, 3-year overall survival rates with CRT in advanced-stage disease, particularly stage IVa, was reported to be low, at approximately 25.9% [13].

In our study, the median OS was 21 months (range, 6–83 months) in the entire cohort. Extended lymph node dissection encompassing both thoracic and abdominal regions was performed in the context of the Ivor Lewis and McKeown esophagectomy procedures. R0 resection was achieved in 101 patients (87.8%) and associated with improved survival in univariate analysis. Multivariate analysis demonstrated that p-Stage IVa was significantly associated with a 5.25-fold increased risk of mortality, whereas p-Stage III–IV was associated with a 2.20-fold increased risk of death, showing a trend toward statistical significance (*p* = 0.028 and *p* = 0.055, respectively). The median EFS and overall survival in the squamous cell group were 18 months and not reached, respectively, while in the adenocarcinoma subgroup they were 8.4 and 12 months, respectively. Survival analyses were stratified by histological subtype (Figure 4 and Figure 5). The median EFS for the entire group was 15 months. The overall recurrence rate was 44%, with lower rates observed in squamous cell carcinoma (39.5%) compared to adenocarcinoma (69.2%). Despite a high proportion of patients with locally advanced disease and ECOG performance status ≥1, as well as a relatively short follow-up duration, which may have led to shorter EFS, the 1-, 3-, and 5-year survival rates remained comparable with the literature [2,5,14].

Although endoscopic biopsy is typically performed shortly after the completion of CRT, due to prominent post-treatment inflammation during this period, false negative or inconclusive results are not uncommon. A second biopsy is generally scheduled 10–14 weeks after CRT completion. If the second biopsy remains inconclusive, patients are excluded from active surveillance protocols and referred for surgery. Likewise, patients with endoscopically non-passable strictures or biopsy-proven dysplasia are not considered suitable candidates for surveillance. In our study, there was no significant difference in EFS between surgical and surveillance patients, and the median OS was not reached (*p* = 0.952 and *p* = 0.955, respectively).

In the literature, OS and disease-free survival were similar between patients who underwent chemoradiotherapy followed by surgery and those managed with active surveillance. No differences were observed between the adenocarcinoma and squamous cell carcinoma subgroups. Although differences in DFS and OS were noted among patients younger than 70, recurrence rates were higher in the active surveillance group. In contrast to other studies, patients aged ≥75 years who achieved a clinical complete response after CRT did not appear to benefit from subsequent surgery [9,15]. Among patients who underwent surgery, the reported 3-year survival rate was 67%; in those who declined surgery and were willing to accept up to a 15% reduction in survival compared with surgical candidates, active surveillance was considered a non-inferior alternative management strategy [16,17,18,19].

### Limitations

This study had several limitations. First, it is a retrospective, single-center analysis, which may limit the generalizability of the findings. Second, since the sample sizes of esophageal squamous cell carcinoma and adenocarcinoma were unequal, the statistical power for subgroup comparisons was limited. Therefore, analyses and treatment outcomes were primarily focused on the squamous cell carcinoma subtype. Although the total number of events was acceptable for survival modeling, some categorical subgroups contained relatively few patients, which may have reduced estimate precision and contributed to wider confidence intervals for certain hazard ratio estimates. Third, while a few early-stage or elderly patients with minimal endoscopic fibrosis were managed with close surveillance, their representation was limited relative to the overall cohort, precluding robust subgroup analysis.

## 5. Conclusions

In patients with non-metastatic, resectable esophageal cancer, histopathological factors such as the absence of LVI, female sex, and SCC histology emerged as significant predictors of both EFS and OS. Notably, stage IVa disease was associated with approximately a fivefold higher risk of mortality compared to other stages. Among those undergoing surgery following CRT, treatment response and survival outcomes were in line with previously published data. A small subgroup of patients who achieved a clinical complete response may be managed with a watch-and-wait strategy based on multidisciplinary oncology board evaluation, particularly among frail patients and those older than 65. Although median survival was not reached in this group, the findings suggest that nonoperative management may be considered in carefully selected patients without clearly compromising long-term outcomes. However, prospective studies with larger patient cohorts are needed to validate these findings.

## Figures and Tables

**Table 1:** Characteristics of patients.

Characteristics	Patients *n* = 155 (%)
Age (mean ± SD = 60.1 ± 12)	
≤65	98 (63.2)
>65	57 (36.8)
Sex	
Male	58 (37.4)
Female	97 (62.6)
Smoking	
Yes	78 (50.3)
No	77 (49.7)
Body mass index	
<19	14 (9.9)
≥19	141 (90.1)
ECOG performance status	
0–1	131 (84.5)
2–3	24 (15.5)
Localization	
Middle	53 (34.2)
Distal	102 (65.8)
Histological type	
Squamous cell	129 (83.2)
Adeno	26 (16.8)
Grade	
1–2	117 (77.0)
3	35 (23.0)
c-T stage	
Tx	2 (1.3)
T1	14 (9.0)
T2	41 (26.5)
T3	73 (47.1)
T4	25 (16.1)
c-N stage	
Nx	76 (49.0)
N1	51 (32.9)
N2	18 (11.6)
N3	10 (6.5)
c-Stage	
1	14 (9.0)
2	65 (41.9)
3	46 (29.7)
4a	30 (19.3)
Surgery	
Yes	112 (72.3)
No	43 (27.7)
Surgery type	
Ivor Lewis	26 (21.3)
Distal esophagectomy total gastrectomy	22 (18.0)
Total esophagectomy McKeown	64 (60.7)
Lymphovascular invasion	
Yes	37 (33.0)
No	75 (67.0)
p-T	
T0	43 (38.1)
T1	21 (18.6)
T2	11 (9.7)
T3	31 (27.4)
T4	7 (6.2)
p-N	
N0	67 (59.3)
N1	3 0 (26.5)
N2	9 (8.0)
N3	7 (6.2)
p-Stage	
0	31 (27.4)
1	21 (18.6)
2	14 (12.4)
3	37 (32.7)
4a	10 (8.8)
Response score	
1	36 (31.9)
2	21 (18.6)
3	36 (31.9)
4	20 (17.6)
Surgical margin	
R0	99 (88.4)
R1	13 (11.6)
Chemotherapy type	
No neoadjuvant	13 (8.3)
Neoadjuvant	142 (91.6)
Adjuvant	
Yes	17 (14.0)
No	105 (86.1)
Radiotherapy	
Concurrent chemoradiotherapy	129 (83.2)
No	20 (12.9)
Only radiotherapy	6 (3.9)
Relapse	
No	86 (55.5)
Yes	69 (44.5)
Alive	93 (60.0)
Dead	62 (40.0)

**Table 2:** Characteristics of patients who achieved a complete clinical response.

Characteristics	Non-Operated Patients with Clinical Complete Response *n* = 16 (%)	Operated Patients with Clinical Complete Response *n* = 16 (%)
Age		
≤65	6 (37.5)	12 (75)
>65	10 (62.5)	4 (25)
ECOG performance status		
0–1	15 (93.7)	15 (93.7)
2	1 (6.3)	1 (6.3)
Localization		
Middle	3 (18.8)	4 (25)
Distal	13 (81.2)	12 (75)
Histological type		
Squamous cell	16 (100)	15 (93.7)
Adeno	0	1 (6.3)
Grade		
1–2	14 (87.5)	15 (93.7)
3	2 (12.5)	1 (6.3)
c-T stage		
T1	2 (12.5)	2 (12.5)
T2	6 (37.5)	5 (31.2)
T3	8 (50.0)	8 (50)
T4	0	1 (6.3)
c-N stage		
Nx	9 (56.2)	10 (62.5)
N1	4 (25.0)	5 (31.2)
N2	4 (18.8)	1 (6.3)
c-Stage		
1	2 (12.5)	2 (12.5)
2	9 (56.3)	9 (56.2)
3	5 (31.2)	4 (25.0)
4a	0	1 (6.3)
Radiotherapy		
Concurrent chemoradiotherapy	16 (100)	15 (93.7)
		1 (6.3)
p-Complete response		
Yes	-	9 (56.3)
No	-	7 (43.7)
Relapse		
No	14 (87.5)	13 (81.2)
Yes	2 (12.5)	3 (18.8)

**Table 3:** Uni and multivariate Cox analyses of characteristic parameters related to event-free survival.

		Univariate Analyses		Multivariate Analyses	
Characteristics	Category	HR (95% CI)	*p*-Value	HR (95% CI)	*p*-Value
Age	≤65 vs. >65	1.44 (0.89–2.34)	0.134		
Sex	Female vs. male	2.47 (1.53–3.97)	<0.001	2.42 (1.22–4.83)	**0.011**
Body mass index	<19 vs. ≥19	0.52 (0.25–1.04)	0.067		
ECOG PS	0–1 vs. 2–3	1.84 (1.03–3.28)	0.037	1.25 (0.50–3.14)	0.630
Histological type	Squamous vs. adeno	2.72 (1.57–4.70)	<0.001	0.98 (0.41–2.31)	0.966
Surgical status	No vs. yes	0.43 (0.26–0.71)	0.001	0.50 (0.00–7.91)	0.985
Pathological response score	1 vs. 2–3–4	3.43 (1.76–6.67)	<0.001	1.19 (0.41–3.45)	0.739
Grade	1–2 vs. 3	2.90 (1.75–4.78)	<0.001	1.24 (0.53–2.90)	0.618
Lymphovascular invasion	No vs. yes	4.95 (2.68–9.14)	<0.001	2.47 (0.84–7.21)	0.098
Resection margin	R0 vs. R1–2	4.05 (1.98–8.25)	<0.001	0.89 (0.32–2.49)	0.832
p-T	0–II vs. III–IV	4.02 (2.19–7.37)	<0.001		
p-N	0 vs. I–III	4.14 (2.21–7.74)	<0.001		
p-Stage	0–II vs. III–IVa	4.09 (2.19–7.66)	<0.001	1.95 (0.90–5.22)	0.052
Adjuvant treatment status	No vs. yes	1.71 (0.79–3.71)	0.169		

ECOG PS, Eastern Cooperative Oncology Group Performance Status; p-T, pathological T stage; p-N, pathological N stage.

**Table 4:** Uni and multivariate Cox analyses of characteristic parameters related to overall survival.

		Univariate Analyses		Multivariate Analyses	
Characteristics	Category	HR (95% CI)	*p*-Value	HR (95% CI)	*p*-Value
Age	≤65 vs. >65	1.61 (0.98–2.67)	0.060		
Sex	Female vs. male	1.89 (1.15–3.12)	0.012	1.47 (0.66–3.25)	0.340
Body mass index	<19 vs. ≥19	0.43 (0.21–0.85)	0.015	0.76 (0.25–2.33)	0.640
ECOG PS	0–1 vs. 2–3	2.23 (1.26–3.96)	0.006	1.46 (0.52–4.03)	0.466
Histological type	Squamous vs. adeno	2.34 (1.30–4.21)	0.004	1.12 (0.41–3.08)	0.820
Surgical status	A vs. yes	0.38 (0.22–0.63)	<0.001	0.57 (0.00–0.93)	0.980
Pathological response score	1 vs. 2–3–4	2.41 (1.21–4.81)	0.012	0.49 (0.10–2.30)	0.372
Grade	1–2 vs. 3	3.02 (1.81–5.03)	<0.001	1.31 (0.55–3.08)	0.534
Lymphovascular invasion	No vs. yes	4.46 (2.34–9.19)	<0.001	3.85 (0.82–17.9)	0.086
Resection margin	R0 vs. R1–2	3.79 (1.77–8.14)	<0.001	0.86 (0.28–2.66)	0.864
p-T	0–II vs. III–IV	3.07 (1.58–5.95)	0.001		
p-N	0 vs. I–III	4.11 (2.02–8.37)	<0.001		
p-Stage	0–II vs. III–IVa	4.08 (2.01–8.31)	<0.001	2.20 (0.80–6.20)	0.055
Adjuvant treatment status	No vs. Yes	0.82 (0.29–2.32)	0.710		

ECOG PS, Eastern Cooperative Oncology Group Performance Status; p-T, pathological T stage; p-N, pathological N stage.

## Data Availability

The data can be made available on logical request.

## Abbreviations

SCC	Squamous cell carcinoma
CRT	Concomitant chemoradiotherapy
FLOT	Fluorouracil, leucovorin, oxaliplatin and docetaxel
CT	Computed tomography
MRI	Magnetic resonance imaging
PET-CT	Positron emission tomography
TNM classification	Tumor, node, and metastasis classification
TRG	Tumor regression grade
*p*CR	Pathological complete response (ypT0N0)
ECOG PS	Eastern Cooperative Oncology Group Performance Status
BMI	Body mass index
LVI	Lymphovascular invasion
EFS	Event-free survival
DFS	Disease-free survival
RFS	Relapse-free survival
OS	Overall survival
